# Construction of a zero-dimensional halide perovskite in micron scale towards a deeper understanding of phase transformation mechanism and fluorescence applications†

**DOI:** 10.1039/d4ra06404f

**Published:** 2024-11-06

**Authors:** Lili Xie, Haiyan Qiu, Yuxin Chen, Yingxue Lu, Yanyan Chen, Lanlan Chen, Shanwen Hu

**Affiliations:** a Department of Health Inspection and Quarantine, School of Public Health, Fujian Medical University Fuzhou Fujian 350122 P.R. China shanwenhu@fjmu.edu.cn; b College of Chemistry, Key Laboratory of Analysis and Detecting Technology, Food Safety MOE, Fuzhou University Fuzhou 350002 Fujian P.R. China

## Abstract

Zero-dimensional (0D) halide perovskites have garnered significant interest due to their novel properties in optoelectronic and energy applications. However, the mechanisms underlying their phase transformations and fluorescence properties remain poorly understood. In this study, we have synthesized a micron-scale 0D perovskite observable under confocal laser scanning microscopy (CLSM). This approach enables us to trace the phase transformation process from 0D to three-dimensional (3D) structures, offering a deeper understanding of the underlying mechanisms. Remarkably, we discovered that this *in situ* transformation is highly sensitive to water, allowing for label-free fluorescent analysis of trace amounts of water in organic solvents through the phase transformation process. Additionally, we have designed a reusable paper strip for humidity analysis leveraging this sensitivity as an application of the micron scale material. Our findings not only elucidate the physicochemical properties of perovskites but also expand the potential of halide perovskite materials in analytical chemistry.

## Abstract 2

A zero-dimensional halide perovskite in micron scale is constructed to gain a deeper understanding of phase transformation mechanism and better fluorescence applications.

## Graphical abstract

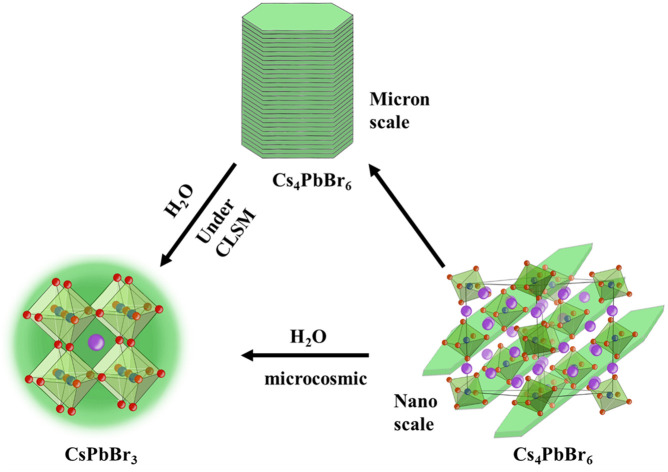

## 1. Introduction

Halide perovskites hold immense potential for next-generation energy materials, sensing substrates and laser devices, with features such as their ultra-high quantum yield, wide color gamut, and excellent optoelectronic properties. The crystal forms of perovskite are closely linked to these characteristics. In 1958, Møller first carried out a complete crystal structure analysis of “colorless” Cs_4_PbX_6_ (X = Cl, Br and I).^1^ In 1999, bulk crystals and thin films of Cs_4_PbX_6_ were fabricated, with their green fluorescence initially attributed to the doping of CsPbBr_3_.^2^ Subsequently, the fluorescence was proposed to be the intrinsic luminescence of Cs_4_PbX_6_.^3^ In 2017, Zhang *et al.* reported the nanocrystalline morphology of Cs_4_PbBr_6_ perovskite for the first time. The prepared colloid and film have high quantum yield (colloid: 65%, film: 54%).^4^ Later, Akkerman *et al.* reported CsPbBr_3_ nanocrystals with almost no light emission, suggesting that their green fluorescence was likely due to trace CsPbBr_3_ undetectable by X-ray diffraction.^5^ Perovskite materials can be used as photocatalysts due to their unique photophysical properties, which have good recyclability.^6^ And by using this property, great achievements have been made in biological imaging.^7^

Pure 0D perovskite is white or colorless regardless of its size while products containing 3D perovskite generally show yellow-green color. Due to its structural instability, Cs_4_PbBr_6_ is easily converted to CsPbBr_3_ under various conditions, such as water, polar solvents, Pb precursors, heating, Prussian blue, and other factors, which can trigger a phase transformation. The phase transition between 0D and 3D perovskite are drawing tremendous attentions.^8,9^ It results in the changes in the crystal structure and physical properties of these materials as a result of variations in temperature, pressure, composition, or other external factors.^10^ Though efforts have been devoted, the mechanisms underlying the phase transition between zero-dimensional and three-dimensional perovskites and origin of the luminescence of Cs_4_PbBr_6_ remain inadequately understood. A variety of theories have been proposed including the migration of ions,^11^ the redistribution of charge,^12^ and the formation of defects,^13^ yet most theories were based on nano-scale perovskite quantum dots, which are challenging to be monitored in real time. The insufficient understanding of mechanisms limits the design and application of the perovskite phase transition.

Among all the notable attributes of perovskites, their fluorescent properties stand out as crucial for various applications.^14^ Perovskites possess a fluorescence quantum efficiency of up to 90%, a tunable wide bandgap within the visible light spectrum, and low exciton binding energy.^15–18^ These properties have significantly propelled advancements in lighting applications, including photovoltaic devices, LED lighting, sensors, and lasers.^19,20^ Furthermore, perovskite-based fluorescence sensing approaches offer the potential for instrument-free visual analysis, particularly for trace water analysis.

This involves monitoring the presence and quality of minute amounts of water in various samples, typically down to parts per million (ppm) or lower.^21–24^ Traditional methods, such as Karl Fischer titration and oven-drying, often fall short in terms of efficiency, convenience, and, more importantly, sensitivity—essential for ensuring that samples meet stringent purity and safety standards. Nonetheless, substantial challenges persist in detecting trace water, such as achieving higher sensitivity and selectivity, reducing false positives, and addressing interference from complex matrices. Conventional approaches that rely on direct reactions between water and signal emitters typically compromise between sensitivity and stability.^21,25,26^ Given the sensitivity of the phase transition between 0D and 3D perovskites to water, the presence of trace amounts of water can significantly influence this transition.^27–29^ The synthesis of micron-scale materials enhances the fluorescence performance associated with this phase transition, thus offering a promising approach for detecting trace amounts of water through phase transformation mechanisms.

Herein we constructed a zero-dimensional perovskite in micrometer scale, which could be witnessed in CLSM with visible fluorescence after transformation. Therefore, the fluorescence changes during the phase transition can be observed in real time, which is beneficial to gain a deep understanding on its phase transition and luminescence mechanism. Furthermore, the fluorescence application of the new materials is expanded to trace water analysis and humidity monitoring.

## 2. Experiment

### 2.1 Reagents and instruments

Ultrapure water, lead bromide (PbBr_2_, 99.0%, Aladdin), cesium carbonate (Cs_2_CO_3_, 99.9%, Aldrich), 1-octadecene (ODE, 90%, Aldrich),oleic acid (OA, 90%, Aldrich), oleyl amine (OAm, 80–90%, Aladdin), *n*-octylamine (98%, Sinopharm), acetic acid (≥99.5%, Sinopharm), *n*-hexane (≥98.0%, Macklin) and other reagent are all purchased from Macklin and used without further purification.

Transmission Electron Microscope (TEM) images were conducted on a Hitachi 6700 transmission electron microscope (Hitachi Ltd, Japan). The fluorescence (FL) measurements were performed on a F-4500 fluorescence spectrophotometer (Hitachi Ltd, Japan). Fluorescence confocal images were recorded by FluoviewSIM-A1 laser scanning confocal microscopy (Nikon Ltd, Japan). Powder X-ray diffraction (XRD) patterns were collected using an Empyrean DY1602 X-ray diffractometer (PAN analytical B.V., the Netherlands).

### 2.2 The synthesis of zero-dimensional perovskite

The synthesis of perovskite is executed in two stages.^30^

Synthesis of Cs precursor: add 0.6516 g of cesium carbonate (CsCO_3_, 2 mmol) and 18 mL of octadecene (ODE), 2 mL of oleic acid (OAc) into a three-neck flask, and heat under a nitrogen atmosphere to 120 °C for 20 minutes, then increase the temperature to 150 °C for another 20 minutes. After the reaction, seal the product in a clean glass bottle to minimize air contact and obtain a 2 mmol mL^−1^ Cs precursor solution that needs to be heated to full dissolution before use.

Perovskite synthesis: in a three-neck flask, add 0.0734 g of lead bromide (PbBr_2_, 0.2 mmol) and 4 mL of octadecene, stir under nitrogen gas, heat to 120 °C, quickly add a certain amount of oleic acid, oleylamine, stearylamine, and acetic acid, and keep for 20 minutes. Then, increase the temperature to a certain level, quickly add 0.5 mL of Cs precursor (0.1 mmol), react for a certain period of time, cool in a water bath, add 20 mL of acetone for centrifugation and wash twice. Put the sample in a glove box to dry at room temperature. After drying the Cs_4_PbBr_6_ powder in the glove box, take 1 mg of it and disperse it in 1 mL of acetone. After enough dispersion, take 20 μL and drop it onto a glass slide or a TEM grid, and let it air-dry.

### 2.3 The phase transformations of zero-dimensional perovskite in micrometer scale

To track the morphological changes and the variations in fluorescence over time, the *n*-hexane-dispersed Cs_4_PbBr_6_ microrods were placed in the air for a predetermined amount of time. Fluorescence confocal images were then taken at an excitation wavelength of 488 nm, as well as the fluorescence values of the samples at various time intervals. Cs_4_PbBr_6_ microrods were dispersed at the same concentration with varying water levels (0.00625%, 0.00833%, 0.01%, and 0.0125%) and the fluorescence signals over time in were recorded.

### 2.4 Trace water analysis

Cs_4_PbBr_6_ at the same concentration with different water contents (0.00625%, 0.00833%, 0.01%, 0.0125%, 0.05%, 0.1%, and 0.2%) were prepared thoroughly mixed, and allowed to react for 150 minutes before the fluorescence spectra were recorded (*λ*_ex_ = 365 nm, *λ*_em_ = 514 nm, slit = 5 nm, PMT = 700 V). For visualization detection, solutions were transferred to sample bottles, arranged in order and photographed under a 365 nm ultraviolet lamp.

For trace water detection using fluorescence confocal microscopy, deionized water in various volumes were added to 300 μL of acetone. Cs_4_PbBr_6_ microrod powder was added to the mixture, and fluorescence confocal microscopy was used to record the kinetics results with the reaction going on.

### 2.5 A paper strip for the humidity analysis

To produce various air humidity settings, cotton and desiccants were put in sealed containers. Cs_4_PbBr_6_ microrod at same volume and concentration were coated on filter paper and placed in various humidity settings. The filter paper's fluorescence photo was measured using a 365 nm UV lamp.

## 3. Results and discussions

### 3.1 The construction of 0D perovskite in micrometer scale

**Scheme 1:**
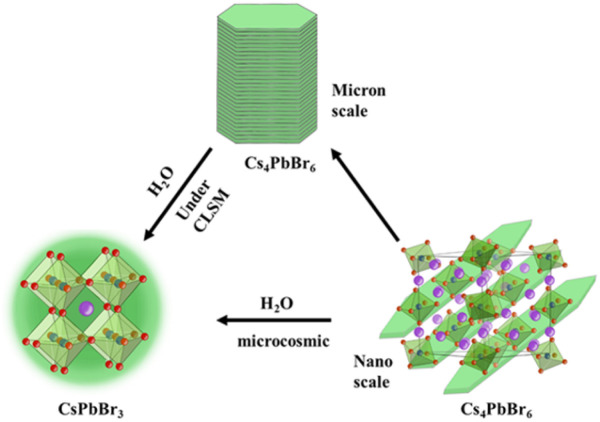
The illustration of the transformation and trace water analysis.

**Fig. 1:**
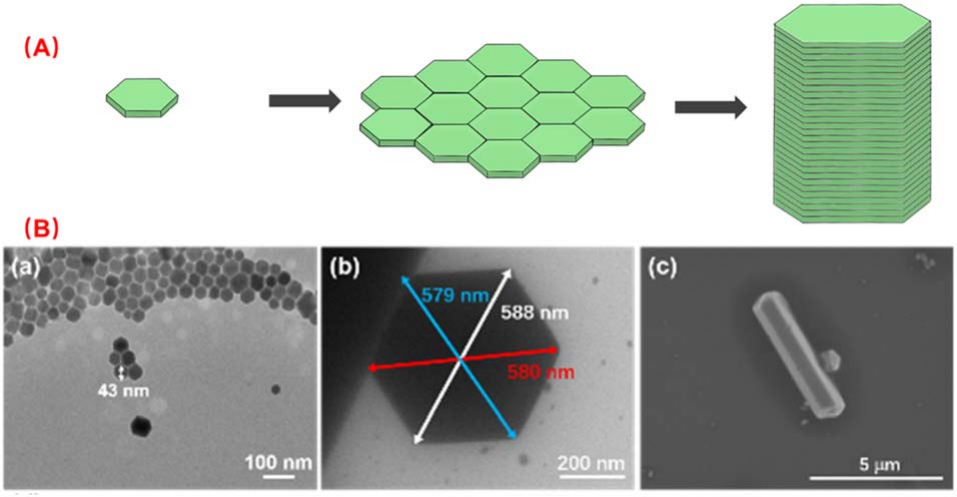
The construction of the 0D perovskite in micrometer scale. (A) Illustration of the two-stage growing from nano scale to micrometer scale. (B) Electron microscope results of the growing process at different time point. (a) TEM image at 10 s, (b) TME image at 20 min, (c) SEM image of the final products.

According to Scheme 1, the nano scale zero dimensional perovskite Cs_4_PbBr_6_ self assembles into micrometer scale zero dimensional perovskite Cs_4_PbBr_6_. When water is present in the environment, cesium bromide in the unstable zero dimensional perovskite Cs_4_PbBr_6_ dissolves, causing [PbBr_6_]^4−^ or Cs in its structure to lack coordination and undergo deformation, leading to dissolution–recrystallization and transformation into structurally stable three-dimensional perovskite CsPbBr_3_. The construction process of 0D Cs_4_PbBr_6_ microrod is illustrated in Fig. 1A. According to the second law of thermodynamics, systems with higher energy states inherently tend to lower their energy spontaneously. In a particle composed of multiple atoms, the surface atoms are less stable than the internal atoms because they form more bonds with surrounding atoms. When many particles aggregate, those with higher surface energy will preferentially connect to reduce overall surface energy, leading to the formation of larger particles. The growth methodology for micron rods leverages this assembly process. In the crystal structure of Cs_4_PbBr_6_, the [PbBr_6_]^4−^ groups are independent and separated by cesium (Cs) atoms, which leads to the instability of the Cs_4_PbBr_6_ crystal structure, resulting in rhombic or hexagonal shape in nano scale. The flat edges of these shapes facilitate the assembly of Cs_4_PbBr_6_. As illustrated in Fig. 1A, the Cs_4_PbBr_6_ nanoplates often appear rhombic or hexagonal, making them more prone to assembly when the edges are flat. The horizontal growth primarily relies on the self-assembly of the outer crystal layer, while the vertical growth, significantly more pronounced than the horizontal, features more ligands on its surface. Acid organic coordinators play a significant role in the vertical growth dynamics, which is highly dependent on the concentration of these organic coordinators and Cs_4_PbBr_6_ nanoplate monomers. Thus, the potential growth method of 0D perovskite nanorods is to extend both horizontal and vertical. Different ligands form chemical interactions with one another over the course of the reaction, progressively assembling nanoplates into corresponding rod-like structures. TEM images highlight the progressive stages of product formation. In Fig. 1B, a TEM image of 43 nm nanoplates are witnessed after 10 seconds of reaction as a preliminary product. Self-assembled disks structures larger than 500 nm are found after 20 minutes horizontal growth. The scanning electron microscopy (SEM) image images of the end products reveal the formation of micron rods following vertical expansion.

### 3.2 The influence factors of the perovskite and phase transformations

The synthesis of CsPbBr_3_ is generally rapidly injected into an ice bath for cooling in a high-temperature environment, while the synthesis of 0D perovskite requires lower temperatures. Due to the instability of its structure, Cs_4_PbBr_6_ is easily converted to CsPbBr_3_, such as water, polar solvents, Pb precursors, heating, Prussian blue, and other factors.^31^ Therefore, we systematically investigated the influence of reaction temperature, reaction time, organic ligand type and amount on the synthesis of micrometer-sized rods of zero-dimensional perovskite.^32,33^

Fig. S1† shows the XRD patterns of perovskite materials synthesized under different conditions. Firstly, the halide perovskite synthesized at different temperature was compared. At 140 °C, the pure Cs_4_PbBr_6_ is formed. When the synthesis occurs at 150 °C, the product remains a white powder of 0D perovskite if the reaction time is under 10 minutes. As reaction time increases, the color gradually darkens, and small peaks of 3D perovskite appear in the X-ray diffraction (XRD) patterns. At 170 °C, the reaction solution quickly turns yellow upon injection of the cesium precursor, and within just 1 minute, the product is a yellow powder comprising a mixture of 3D and 0D perovskite. The XRD of products shown in Fig. S1† reveals the differences brought by the temperature. Thus, to synthesize pure zero-dimensional halide perovskite, a reaction temperature of 140 °C must be maintained.

A sufficient reaction time is crucial for the growth of nanoparticles into microscale rods. With increasing reaction time, small unassembled perovskite particles progressively disappear, leading to more uniform micrometer-sized rods. However, prolonged reaction times can cause particle breakdown and agglomeration or form elongated structures, as reported in the literature.^34^ Therefore, to synthesize regular 0D micrometer rods, the reaction time must be controlled within an optimal range. Herein 20 minutes is chosen.

Different ligands play vital roles in functionalization and stability of nano materials. Commonly used oil-phase ligands are long-chain organic molecules, such as organic compounds with different carbon chain lengths of amides, organic compounds containing N, P, O, S and other elements with strong bonding ability. Adding an appropriate amount of ligand during the synthesis process not only dissolves the solid precursor, improves the reaction activity and efficiency, but also controls the growth rate of the material, thus affecting the size and shape of the product. After the synthesis, the adsorption of the ligand on the surface of the material can improve the stability and dispersibility. We compared five common ligands in the synthesis (listed in Table S1†). Oleic acid and oleylamine are long-chain ligands with 18 carbon atoms, octanoic acid and octyl amine are relatively long-chain ligands with 8 carbon atoms, while ethylic acid is a short-chain and highly active ligand. Different combinations of ligands will have different impacts on the type and morphology of products.^35,36^ In reported literature, the combination of oleic acid and oleylamine can determine if the product is 0D or 3D in nanoscale.^37^ In an oleic acid environment, layered CsPbBr_3_ perovskite can self-assemble into long shapes,^38^ but excessive oleylamine results in the by-product Cs_4_PbBr_6_. The combination of oleic acid, oleylamine, octanoic acid, and octylamine with a small amount of Pb and Cs precursors can synthesize nanoscale CsPbBr_3_ or even micron-scale thin films with uniform size and high luminous efficiency. Octylamine and acetic acid can help to generate micron-scale CsPbBr_3_ films.

Using oleic acid and oleylamine can ensure the synthesis of micrometer-sized rods, however, excessive long-chain oleic acid will increase the instability of Cs_4_PbBr_6_, facilitating its transformation. Therefore, adding a corresponding short-chain acid can help expand the length into micrometer-sized rods while avoiding undesirable transformations during synthesis. The type of ligand determines the material morphology and size changes with the amount of ligand used. Herein, the Pb : Cs ratio was fixed at 2 : 1, reaction temperature was 140 °C, and reaction time was 20 minutes. We varied the amount of oleic, stearic, and succinic acid and the results were listed in Table S2,† the corresponding TEM images are shown in Fig. S2.† As the amount of acid increases, the length in the longitudinal direction will increase, and the increase of amines will correspondingly broaden the width in the transverse direction. However, a high proportion of amines leads to a higher yield of rod-shaped perovskite products, which is not favorable for synthesizing rod-shaped 0D perovskite. When the acid-amine ratio exceeds 3, a large amount of 3D perovskite nanocrystals with small particles will be produced, and the composition of the three-dimensional perovskite will increase.

### 3.3 The phase transformations of zero-dimensional perovskite in micrometer scale

**Fig. 2:**
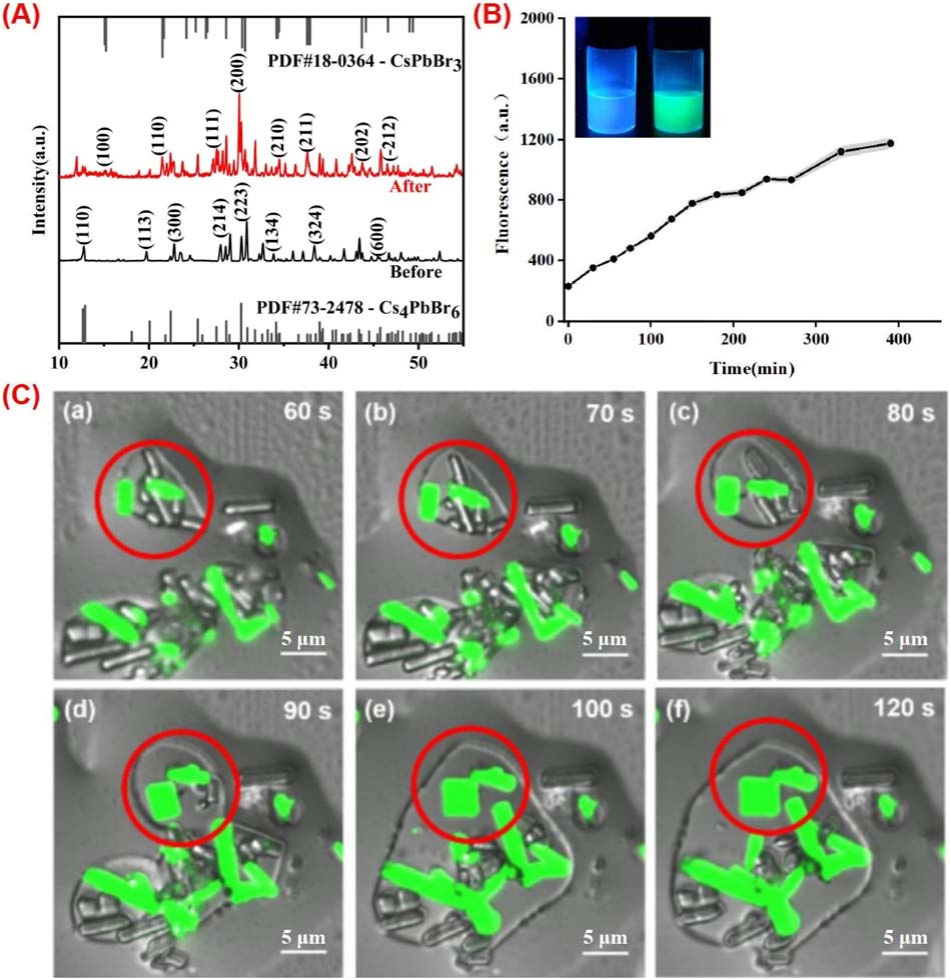
The phase transformation process between 0D and 3D. (A) XRD results of the 0D and 3D perovskite. (B) Fluorescent photograph of 0D and 3D under UV light and fluorescence intensity of the transformation. (C) Confocal diagram of fluorescence of Cs_4_PbBr_6_ microrods over time after 60 s guided by water.

The phase transformation process is essential for the perovskite-based analysis.^39^ The XRD spectra of 0D and 3D perovskite are shown in Fig. 2A. The characteristic peaks of Cs_4_PbBr_6_ are revealed at 2*θ* = 12.9°, 20.1°, 22.4°, 28.6°, 30.3°, 33.1°, 38.9° and 45.7°, corresponding to diffractions from (110), (113), (300), (214), (223), (134), (324) and (600) crystal planes of the rhombohedral Cs_4_PbBr_6_ phase. These diffraction peaks are consistent with the standard XRD pattern of Cs_4_PbBr_6_ (PDF card #73-2478). Indicating that the main product is Cs_4_PbBr_6_. After the transformation induced by adding a certain amount of water to Cs_4_PbBr_6_ solution, the diffraction peaks of CsPbBr_3_ (PDF card #18-0364) were observed at 15.2°, 21.5°, 26.4°, 30.7°, 34.1°, 37.6°, and 43.7°, indexed to (100), (110), (111), (200), (210), (211) and (202) crystal planes, indicating the phase transformation from Cs_4_PbBr_6_ to CsPbBr_3_.

Despite the large differences in crystal structure, the phase transition between them is easily achieved under certain physical and chemical conditions, usually triggered by the chemical stripping of bromide cesium from Cs_4_PbBr_6_.^40^ For example, 0D can be converted to 3D through thermal annealing or chemical reaction with Prussian blue reagents, as reported in the literature. There are also suggestions that 0D nano polyhedral can convert into CsPbBr_3_ through the help of the TOAB ligand.^41^ Since the 0D perovskite was successfully synthesized in micro scale, the transformation process is notably more observable.

The enormous rod-like morphology of the synthetic zero-dimensional perovskite microrods allows us to directly see the morphological changes using CLSM. The zero-dimensional microrods themselves do not emit light, however as the applied conditions change, corresponding fluorescence changes can be obtained (Fig. 2B). This enables *in situ* observation of the transition from 0D to 3D perovskite, which can reveal the luminescence mechanism more deeply.

Water has a substantial effect on the 0D perovskite because it can dissolve the cesium bromide in the powder, turning the Cs-rich 0D structure into 3D structure. A potential mechanism for the phase transformation is dissolution–reprecipitation process, which can be confirmed by size enlargement. However, it is not feasible to observe the dynamic enlargement at the nanoscale, the size of the quantum dots rarely changes after the transformation. Herein, in micro scale, from the non-emissive Cs_4_PbBr_6_ microrods at initial, the *in situ* monitoring of the transition is revealed in Fig. 2C. The undamaged microrods will dissolve and grow thinner after 60 seconds of adding water. When water surrounds on the surface of the substance and dissolves cesium bromide. The monomers that have been dissolved will spread across the environment and 3D CsPbBr_3_ appears then. The CsPbBr_3_ inside the red circle gradually coalesces with the surrounding monomers, expands again until all of the monomers are consumed, resulting in strongly emissive rods or plates. Notably, we can draw the conclusion that the transition involves a dissolution–reprecipitation process. The real-time alterations occurring during the transformation process were therefore seen. Furthermore, we can deduce that water not only dissolves cesium bromide in Cs_4_PbBr_6_ crystal, but also undergoes phase transition and breaks down pre-assembled microrods into tiny CsPbBr_3_ monomers, which reassemble into particles with various forms.

### 3.4 Trace water analysis based on the phase transformation

**Fig. 3:**
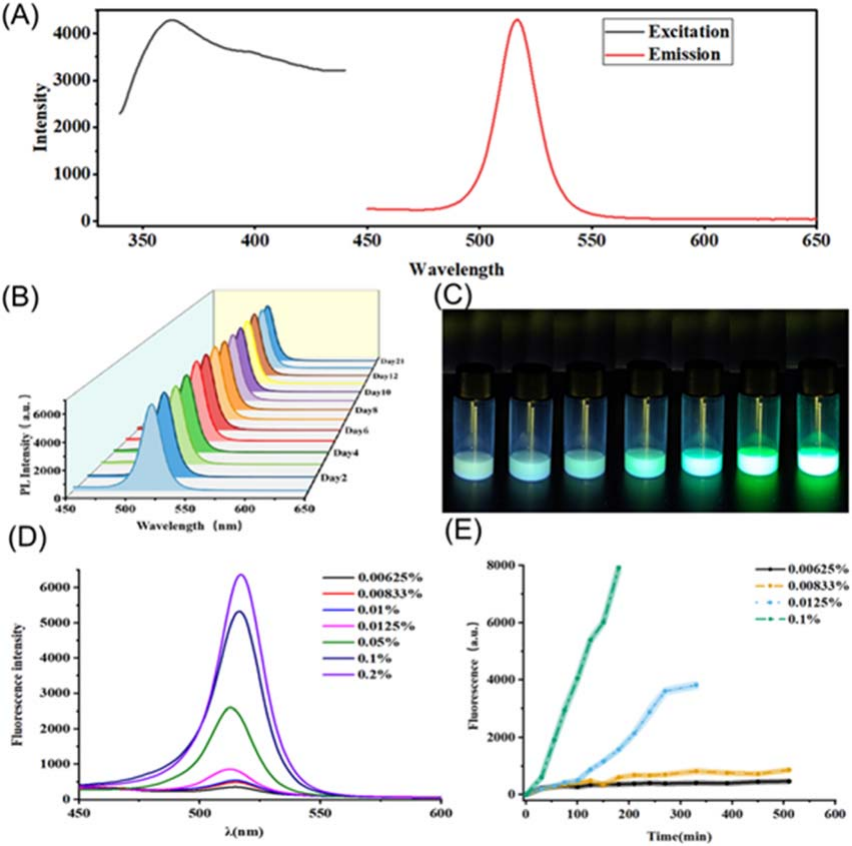
Trace water analysis by the transformation process. (A) The excited and emitted spectra of the 3D perovskite. (B) The fluorescent signal changes with time. (C) The fluorescent photograph under UV light with different content of water. Water content from left to right: 0.00625%, 0.0083%, 0.01%, 0.0125%, 0.1%, 0.2%. (D) The fluorescence spectra with different content of water. (E) The fluorescence intensity during transformation process with different water content.

The water-induced transformation process offers an innovative approach for trace water analysis. We applied the prepared materials to microscale trace water analysis. The fluorescence spectra of the 3D perovskite are shown in Fig. 3A, with an excitation wavelength of 365 nm and an emission peak at 516 nm. This signal demonstrates excellent time stability, with less than a 5% loss over 21 days, as illustrated in Fig. 3B. In complex chemical environments, the stable fluorescence signal can prevent inaccuracies caused by environmental fluctuations, which often affect traditional methods. By utilizing millimeter-scale perovskites, the water-induced phase transformation process can be real-time monitored through changes in fluorescence over a short period and allows for quantitative detection based on the fluorescence signal intensity. As shown in Fig. 3C, the transformation speed accelerates with increasing water content. At the same time intervals, the resulting products exhibit varying fluorescence intensities. As depicted in Fig. 3D, the fluorescence peaks heighten with increased water content. Noticeable differences can be observed during the transformation process, indicating its potential for visualized trace water sensing, as illustrated in Fig. 3E.

### 3.5 Humidity analysis on paper

The transformation process can also be triggered by the airborne water. The micro scale 0D materials allowed us to explore this transversion *via* CLSM. The fluorescence variations of Cs_4_PbBr_6_ microrods exposed to air for various durations are shown in Fig. S3† Within 30 seconds, the smaller particles were the first to emit light. A higher specific surface area leads to comparatively larger interaction areas with airborne water molecules, which induces a rapid phase change and fluorescence emission. Subsequently, some edges begin to emit light. Eventually, Cs_4_PbBr_6_ starts to break down due to the environment's water molecules dissolving cesium bromide. The resulting particles from the breakdown of several microrods then aggregate to form CsPbBr_3_ nanocrystals and emit light. Consequently, the majority of the emitting particles in Fig. S3e and f† are located at the microrod connections.

**Fig. 4:**
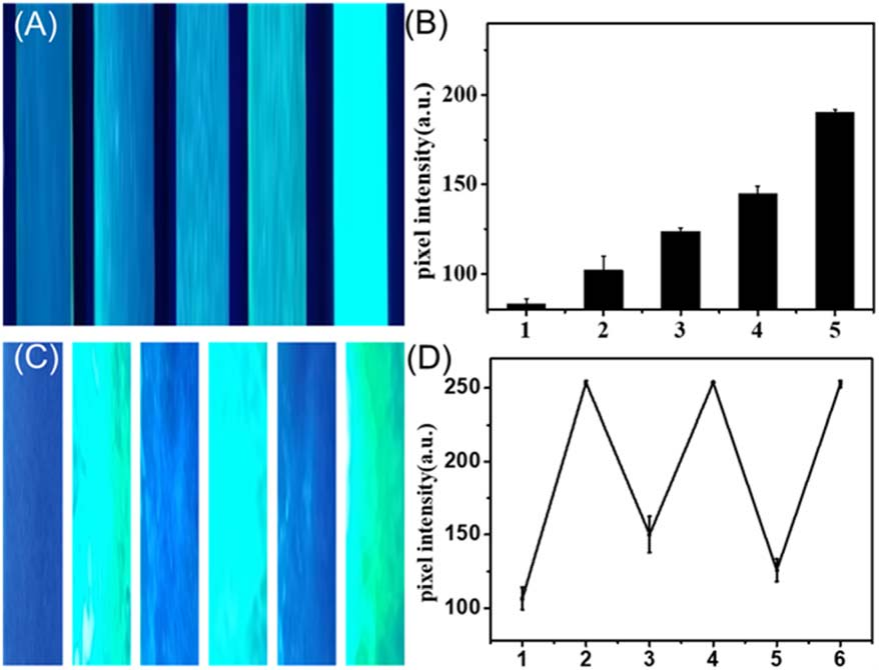
Humidity analysis on paper. (A) Fluorescent photograph of paper under UV light with different humidity (Cs_4_PbBr_6_ humidity sensitive test strips were placed from left to right at distances of 50 cm, 20 cm, 15 cm, 10 cm, and 5 cm from the humidifier, with the relative humidity gradually increasing). (B) The pixel analysis results of paper with different humidity. (C) Fluorescent photograph of paper with cyclic humidity sensing under UV light. (D) The pixel analysis results of paper during cyclic humidity sensing.

To demonstrate its superiority for on-site and real-time water sensing, we deposit the 0D perovskite on paper substrate to get a humidity analysis platform. The paper was exposed in air with varying relative humidity levels, which triggered the transformation process, resulting in visible fluorescence signals on the paper. As can be witnessed in Fig. 4A, fluorescence photographs taken under UV lamp excitation exhibit a significant positive correlation between fluorescence intensity and relative humidity. The quantitative results of pixel intensity analysis for the green colorimetry of each test strip, as illustrated in Fig. 4B, also demonstrate consistency.

The dissolution–reprecipitation mechanism revealed under CLSM inspired us to construct a reversible transformation process. Interestingly, we witnessed that the 3D perovskite can slowly transform back to its 0D phase without fluorescence in a vacuum-drying environment. This reversible process turns the test strip into a reusable cyclic responsive paper. As displayed in Fig. 4C (pixel intensity shown in Fig. 4D), we utilized the same strip to expose to moisture-laden air, reset it, and then respond again, repeating this process three times and resetting it twice. We obtained stable results, indicating that this cyclic response capability is reliable, which reduced the cost of the test strip. The possible mechanism is, the 3D perovskite generated through the dissolution–recrystallization process gradually loses its crystallization water under dry conditions, leading to the disruption of the crystalline structure and reverting to its original state. This paper-based reversible detection offers a simple, portable and cost-effective option for humidity monitoring.

## 4. Conclusion

A stable micro scale 0D perovskite has been successfully constructed, enabling observation under an optical microscope. A dissolution–reprecipitation process is monitored on-site in real-time under CLSM, providing a deeper understanding of the mechanism behind the phase transition of 0D perovskite. Furthermore, this larger-sized perovskite exhibited stronger intrinsic fluorescence, and we utilized the water-induced phase transition process to detect trace amounts of water in organic solvents. Moreover, we fabricated a humidity sensing paper using this material, which demonstrated sensitive, stable, and reversible sensing capacity of humidity in the air. We believe that this micro scale perovskite holds great potential in both theoretical research and practical applications.

## Data availability

The data that support the findings of this study are available on request from the corresponding author.

## Author contributions

Lili Xie and Haiyan Qiu: writing – original draft, data curation, investigation. Yuxin Chen and Yanyan Chen: formal analysis, investigation, validation. Yingxue Lu: validation. Lanlan Chen: investigation, writing – review & editing. Shanwen Hu: conceptualization, project administration, writing – review & editing.

## Conflicts of interest

The authors declare no competing financial interest.

## Supplementary Material

RA-014-D4RA06404F-s001

## References

[cit1] MØller C. K. (1958). Crystal Structure and Photoconductivity of Cæsium Plumbohalides. Nature.

[cit2] Nikl M., Mihokova E., Nitsch K., Somma F., Giampaolo C., Pazzi G. P., Fabeni P., Zazubovich S. (1999). Photoluminescence of Cs_4_PbX_6_ crystals and thin films. Chem. Phys. Lett..

[cit3] Saidaminov M. I., Almutlaq J., Sarmah S., Dursun I., Zhumekenov A. A., Begum R., Pan J., Cho N., Mohammed O. F., Bakr O. M. (2016). Pure Cs_4_PbBr_6_: Highly Luminescent Zero Dimensional Perovskite Solids. ACS Energy Lett..

[cit4] Zhang Y. H., Saidaminov M. I., Dursun I., Yang H. Z., Murali B., Alarousu E., Yengel E., Alshankiti B. A., Bakr O. M., Mohammed O. F. (2017). Zero-Dimensional Cs_4_PbBr_6_ Perovskite Nanocrystals. J. Phys. Chem. Lett..

[cit5] Akkerman Q. A., Park S., Radicchi E., Nunzi F., Mosconi E., De Angelis F., Brescia R., Rastogi P., Prato M., Manna L. (2017). Nearly Monodisperse Insulator Cs_4_PbX_6_ (X = Cl, Br, I) Nanocrystals, Their Mixed Halide Compositions, and Their Transformation into CsPbX_3_ Nanocrystals. Nano Lett..

[cit6] Zhu Y. F., Zhang J. H. (2024). Antimony-Based Halide Perovskite Nanoparticles as Lead-Free Photocatalysts for Controlled Radical Polymerization. Macromol. Rapid Commun..

[cit7] Zhang J. H., Zhu Y. F. (2024). Exploiting the Photo-Physical Properties of Metal Halide Perovskite Nanocrystals for Bioimaging. Chembiochem.

[cit8] Cao F., Yu D. J., Xu X. B., Han Z. Y., Zeng H. B. (2021). CsPbBr_3_@Cs_4_PbBr_6_ Emitter-in-Host Composite: Fluorescence Origin and Interphase Energy Transfer. J. Phys. Chem. C.

[cit9] Mao Y. L., Zhang J., Ren Q. Q., Molokeev M. S., Zhou G. J., Zhang X. M. (2022). Unveiling the uncommon blue-excitable broadband yellow emission from self-trapped excitons in a zero-dimensional hybrid tellurium-based double perovskite. J. Mater. Chem. C.

[cit10] Shellaiah M., Sun K. W., Thirumalaivasan N., Bhushan M., Murugan A. (2024). Sensing Utilities of Cesium Lead Halide Perovskites and Composites: A Comprehensive Review. Sensors.

[cit11] Van Der Stam W., Geuchies J. J., Altantzis T., Van Den Bos K. H. W., Meeldijk J. D., Van Aert S., Bals S., Vanmaekelbergh D., Donega C. D. (2017). Highly Emissive Divalent-Ion-Doped Colloidal CsPb_1-x_M_x_Br_3_ Perovskite Nanocrystals through Cation Exchange. J. Am. Chem. Soc..

[cit12] Xie J. L., Huang Z. Q., Wang B., Chen W. J., Lu W. X., Liu X., Song J. L. (2019). New lead-free perovskite Rb_7_Bi_3_Cl_16_ nanocrystals with blue luminescence and excellent moisture-stability. Nanoscale.

[cit13] Liu H. W., Wu Z. N., Shao J. R., Yao D., Gao H., Liu Y., Yu W. L., Zhang H., Yang B. (2017). CsPb_x_Mn_1-x_Cl_3_ Perovskite Quantum Dots with High Mn Substitution Ratio. ACS Nano.

[cit14] He J., Xu X. M., Li M. S., Zhou S. Y., Zhou W. (2023). Recent advances in perovskite oxides for non-enzymatic electrochemical sensors: A review. Anal. Chim. Acta.

[cit15] Kojima A., Teshima K., Shirai Y., Miyasaka T. (2009). Organometal Halide Perovskites as Visible-Light Sensitizers for Photovoltaic Cells. J. Am. Chem. Soc..

[cit16] Lee M. M., Teuscher J., Miyasaka T., Murakami T. N., Snaith H. J. (2012). Efficient Hybrid Solar Cells Based on Meso-Superstructured Organometal Halide Perovskites. Science.

[cit17] Ma Z. H., Li X. D., Zhang C. X., Turyanska L., Lin S., Xi X., Li J., Hu T. G., Wang J. F., Patanè A., Zhao L. X. (2021). CsPb(Br/I)_3_ Perovskite Nanocrystals for Hybrid GaN-Based High-Bandwidth White Light-Emitting Diodes. ACS Appl. Nano Mater..

[cit18] Hai O., Li J., Pei M. K., Ren Q., Wu X. L., Qin B., Xiao X. N., He X. L., Li T., Chen Y. (2023). Perovskite-Based Nanostructures for Fluorescence Afterglow Anticounterfeiting. ACS Appl. Nano Mater..

[cit19] Ju M. G., Dai J., Ma L., Zhou Y. Y., Zeng X. C. (2018). Zero-Dimensional Organic-Inorganic Perovskite Variant: Transition between Molecular and Solid Crystal. J. Am. Chem. Soc..

[cit20] Cho H. B., Min J. W., Kim H., Viswanath N. S. M., Samanta T., Han J. H., Park Y. M., Jang S. W., Im W. B. (2022). Three-Dimensional Lead Halide Perovskites Embedded in Zero-Dimensional Lead Halide Perovskites: Synthesis, Stability, and Applications. ACS Appl. Electron. Mater..

[cit21] Zhou L., Liao J. F., Huang Z. G., Wei J. H., Wang X. D., Li W. G., Chen H. Y., Kuang D. B., Su C. Y. (2019). A Highly Red-Emissive Lead-Free Indium-Based Perovskite Single Crystal for Sensitive Water Detection. Angew. Chem., Int. Ed..

[cit22] Wang J., Huang Y. J., Gao Z. Q., Du J. X. (2024). Lead-Free Antimony-Based Perovskite Nanocrystals as a Fluorescent Nanoprobe for the Turn-On Detection of Trace Water in Organic Solvents. ACS Appl. Nano Mater..

[cit23] Wu Z. L., Yang J., Sun X., Wu Y. J., Wang L., Meng G., Kuang D. L., Guo X. Z., Qu W. J., Du B. S., Liang C. Y., Fang X. D., Tang X. S., He Y. (2021). An excellent impedance-type humidity sensor based on halide perovskite CsPbBr_3_ nanoparticles for human respiration monitoring. Sens. Actuators, B.

[cit24] Ju Y. Y., Peng J., Chen Y., Huang S., Gu K., Cai S. S., Huang L. L., Zhong H. Z. (2022). Water-Sensitive Mixed-Phase PEA_6_SnI_8_ Perovskite Derivative Single Crystal for Humidity Detection. Cryst. Growth Des..

[cit25] Wang S., Yu L., Wei Z. Y., Xu Q., Zhou W. B., Xiao Y. X. (2022). Dual-response ratiometric fluorescence based ligand-functionalized CsPbBr_3_ perovskite quantum dots for sensitive detection of trace water in edible oils. Sens. Actuators, B.

[cit26] Li Z., Chen X. L., Yu L., Li H. J., Chen L., Kang Q., Shen D. Z. (2020). Monitoring of reaction kinetics and determination of trace water in hydrophobic organic solvents by a smartphone-based ratiometric fluorescence device. Microchim. Acta.

[cit27] Guo R. J., Xiong Q., Ulatowski A., Li S. S., Ding Z. J., Xiao T. X., Liang S. Z., Heger J. E., Guan T. F., Jiang X. Y., Sun K., Reb L. K., Reus M. A., Chumakov A., Schwartzkopf M., Yuan M. J., Hou Y., Roth S. V., Herz L. M., Gao P., Müller-Buschbaum P. (2024). Trace Water in Lead Iodide Affecting Perovskite Crystal Nucleation Limits the Performance of Perovskite Solar Cells. Adv. Mater..

[cit28] Zhang M. Q., Bi C. H., Xia Y. X., Sun X. J., Wang X. Y., Liu A. Q., Tian S. Y., Liu X. F., De Leeuw N. H., Tian J. J. (2023). Water-Driven Synthesis of Deep-Blue Perovskite Colloidal Quantum Wells for Electroluminescent Devices. Angew. Chem., Int. Ed..

[cit29] Xiang X. X., Ouyang H., Li J. Z., Fu Z. F. (2021). Humidity-sensitive CsPbBr_3_ perovskite based photoluminescent sensor for detecting Water content in herbal medicines. Sens. Actuators, B.

[cit30] Yang L., Li D. M., Wang C., Yao W., Wang H., Huang K. X. (2017). Room-temperature synthesis of pure perovskite-related Cs_4_PbBr_6_ nanocrystals and their ligand-mediated evolution into highly luminescent CsPbBr_3_ nanosheets. J. Nanopart. Res..

[cit31] Liang W. C., Li T., Zhu C. C., Guo L. D. (2022). Ligand-mediated evolution from CsPbBr_3_ to Cs_4_PbBr_6_/CsPbBr_3_ perovskite composites with intense green emission. Optik.

[cit32] Rao L. S., Ding X. R., Du X. W., Liang G. W., Tang Y., Tang K., Zhang J. Z. (2019). Ultrasonication-assisted synthesis of CsPbBr_3_ and Cs_4_PbBr_6_ perovskite nanocrystals and their reversible transformation. Beilstein J. Nanotechnol..

[cit33] Huang C. Y., Huang S. H., Wu C. L., Wang Z. H., Yang C. C. (2020). Cs_4_PbBr_6_/CsPbBr_3_ Nanocomposites for All-Inorganic Electroluminescent Perovskite Light-Emitting Diodes. ACS Appl. Nano Mater..

[cit34] Zhang D. D., Yang Y. M., Bekenstein Y., Yu Y., Gibson N. A., Wong A. B., Eaton S. W., Kornienko N., Kong Q., Lai M. L., Alivisatos A. P., Leone S. R., Yang P. D. (2016). Synthesis of Composition Tunable and Highly Luminescent Cesium Lead Halide Nanowires through Anion-Exchange Reactions. J. Am. Chem. Soc..

[cit35] Pan A. Z., He B., Fan X. Y., Liu Z. K., Urban J. J., Alivisatos A. P., He L., Liu Y. (2016). Insight into the Ligand-Mediated Synthesis of Colloidal CsPbBr_3_ Perovskite Nanocrystals: The Role of Organic Acid, Base, and Cesium Precursors. ACS Nano.

[cit36] Sun S. B., Yuan D., Xu Y., Wang A. F., Deng Z. T. (2016). Ligand-Mediated Synthesis of Shape-Controlled Cesium Lead Halide Perovskite Nanocrystals *via* Reprecipitation Process at Room Temperature. ACS Nano.

[cit37] Liang Z. Q., Zhao S. L., Xu Z., Qiao B., Song P. J., Gao D., Xu X. R. (2016). Shape-Controlled Synthesis of All-Inorganic CsPbBr_3_ Perovskite Nanocrystals with Bright Blue Emission. ACS Appl. Mater. Interfaces.

[cit38] Mehetor S. K., Ghosh H., Pradhan N. (2019). Acid-Amine Equilibria for Formation and Long-Range Self-Organization of Ultrathin CsPbBr_3_ Perovskite Platelets. J. Phys. Chem. Lett..

[cit39] Li L. J., Zhang Z. H. (2022). In-situ fabrication of Cu doped dual-phase CsPbBr_3_-Cs_4_PbBr_6_ inorganic perovskite nanocomposites for efficient and selective photocatalytic CO_2_ reduction. Chem. Eng. J..

[cit40] Palazon F., Urso C., De Trizio L., Akkerman Q., Marras S., Locardi F., Nelli I., Ferretti M., Prato M., Manna L. (2017). Postsynthesis Transformation of Insulating Cs_4_PbBr_6_ Nanocrystals into Bright Perovskite CsPbBr_3_ through Physical and Chemical Extraction of CsBr. ACS Energy Lett..

[cit41] Jing Q., Su Y. C., Xing X., Lu Z. D. (2019). Highly luminescent CsPbBr_3_ nanorods synthesized by a ligand-regulated reaction at the water-oil interface. J. Mater. Chem. C.

