# Supplementary material for: Construction of a zero-dimensional halide perovskite in micron scale towards a deeper understanding of phase transformation mechanism and fluorescence applications
Source: RSC Adv. 2024 Nov 6;14(48):35490–7. doi: 10.1039/d4ra06404f (PMC11539010; doi:10.1039/d4ra06404f)
Supplement: RA-014-D4RA06404F-s001 [file RA-014-D4RA06404F-s001.pdf]

Supplementary Information (SI) for RSC Advances.
This journal is © The Royal Society of Chemistry 2024

Supporting Information

Construction of Zero-Dimensional Halide Perovskite in Micron Scale towards a Deeper Understanding of Phase Transformations Mechanism and Fluorescent applications

Lili Xie,[#a] Haiyan Qiu,[#a] Yuxin Chen,[a] Yingxue Lu,[a] Yanyan Chen,[b] Lanlan Chen,[b] Shanwen Hu[*a]

a. Department of Health Inspection and Quarantine, School of Public Health, Fujian Medical University, Fuzhou, Fujian, 350122, P.R. China.

b. College of Chemistry, Key Laboratory of Analysis and detecting technology, Food Safety MOE, Fuzhou University, Fuzhou 350002, Fujian, P.R. China.

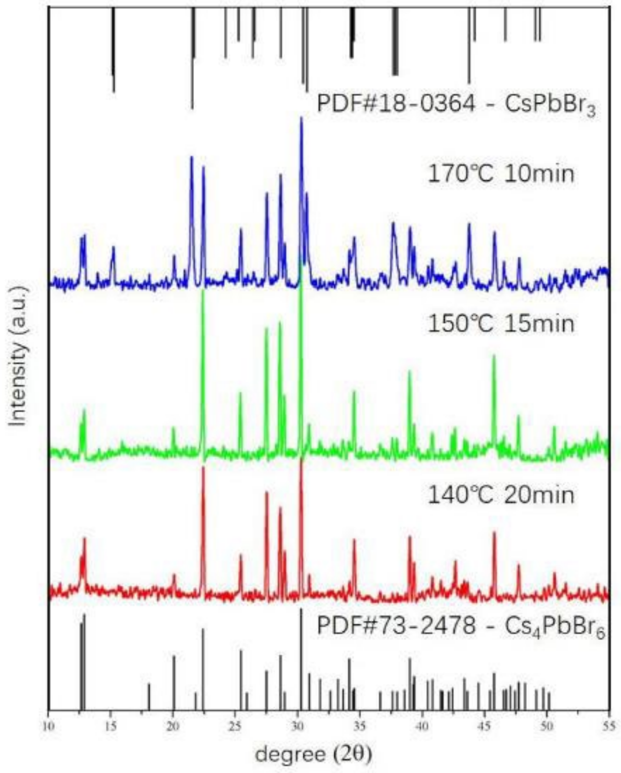

Fig. S1 XRD spectra of Cs_4PbBr_6 synthesized under different reaction conditions.

Table. S1 Information of ligands used in the synthesis

oleic acid	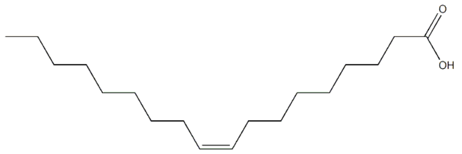
Oleyl amine	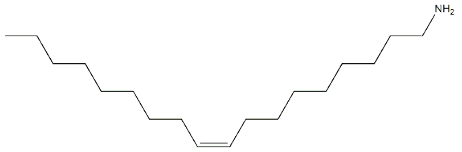
octoic acid	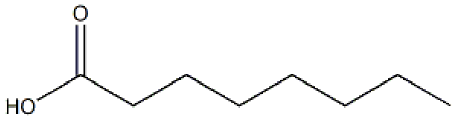
octyl amine	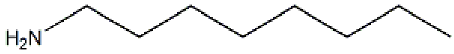
ethylic acid	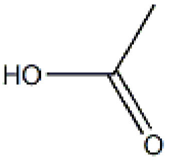

Table. S2 The size of products with different ratio of ligands

oleic acid (mL)	Oleylamine (mL)	octyl amine (mL)	ethylic acid (mL)	Products size
0.8	0.8	0.8	0	(0.5~1.0 μm)*400 nm
0.8	0.8	0.8	0.1	(0.8~1.4 μm)*400 nm
0.8	0.8	0.8	0.4	(2.5~4 μm)*400 nm
0.6	0.8	0.8	0.4	(2.5~3 μm)*400 nm
1.2	0.8	0.8	0.4	(6~7 μm)*400 nm
0.8	0.8	0.6	0.4	500 nm*500 nm
0.8	0.8	1.2	0.4	20 μm *500 nm
0.8	0.6	0.8	0.4	2.5 μm *2.5 μm
0.8	1.2	0.8	0.4	2.27 μm *300 nm
1.4	0.8	0.8	0.6	(9~10 μm)*700 nm

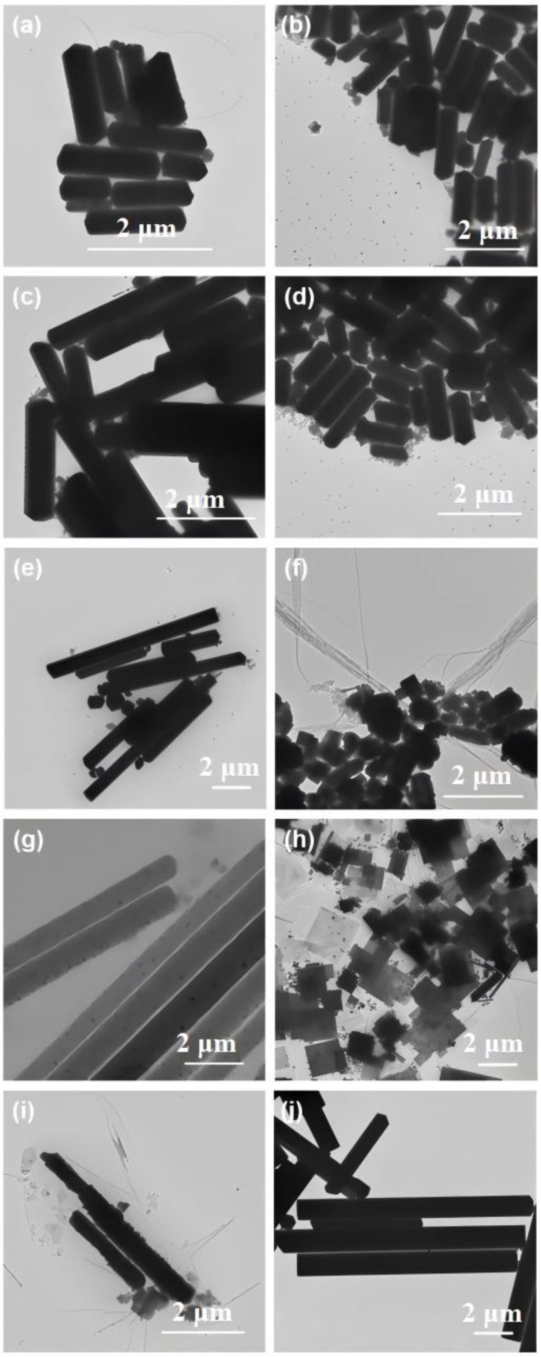

Fig. S2 TEM images of Cs_4PbBr_6 synthesized with different ratio of ligands.

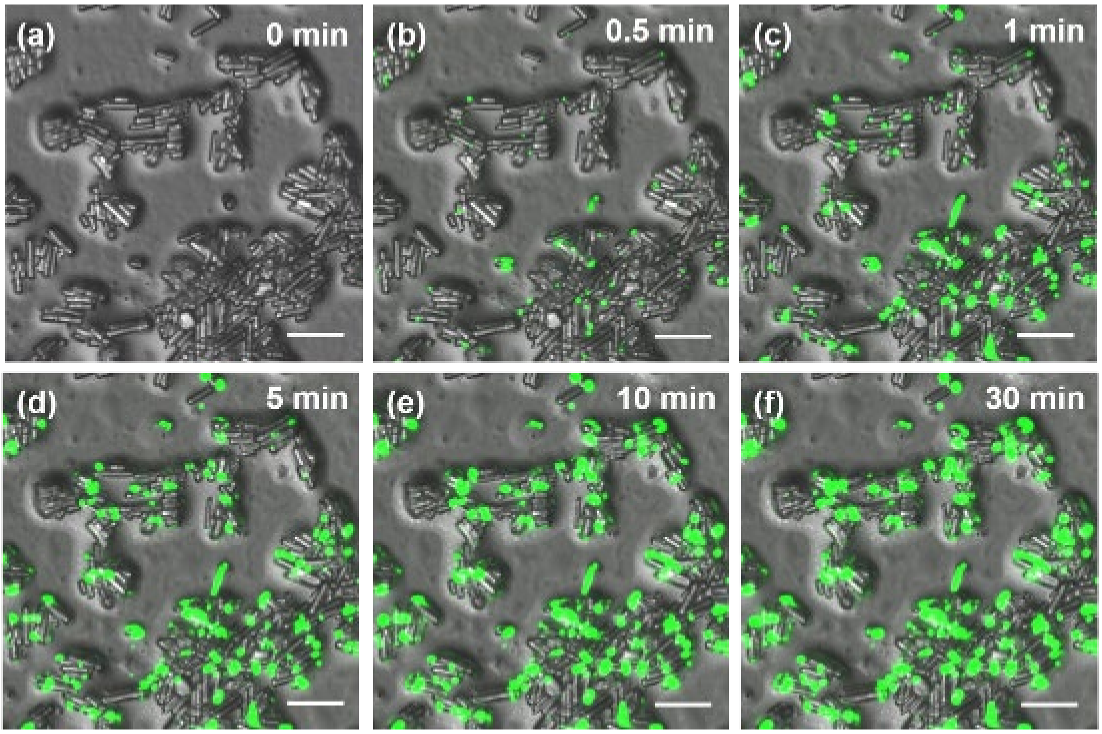

Fig. S3 CLSM image of micro scale Cs_4PbBr_6 with airborne water induced transformation. The legend is 10 μm, with excited wavelength 488 nm.
